# Determination of stable reference genes for RT-qPCR expression data in mechanistic pain studies on pig dorsal root ganglia and spinal cord

**DOI:** 10.1016/j.rvsc.2017.09.025

**Published:** 2017-10

**Authors:** Dale A. Sandercock, Jennifer E. Coe, Pierpaolo Di Giminiani, Sandra A. Edwards

**Affiliations:** a Animal and Veterinary Science Research Group, Scotland's Rural College (SRUC), West Mains Road, Edinburgh EH16 4SA, UK; b School of Agriculture, Food and Rural Development, Newcastle University, Newcastle upon Tyne NE1 7RU, UK

**Keywords:** Pig, RT-qPCR, Dorsal root ganglia, Spinal cord, Reference genes, Normalisation

## Abstract

RNA expression levels for genes of interest must be normalised with appropriate reference or “housekeeping” genes that are stably expressed across samples and treatments. This study determined the most stable reference genes from a panel of 6 porcine candidate genes: beta actin (*ACTB*), beta-2-microglobulin (*B2M*), eukaryotic elongation factor 1 gamma-like protein (*eEF-1*), glyceraldehyde-3-phosphate dehydrogenase (*GAPDH*), succinate dehydrogenase complex subunit A (*SDHA*), Ubiquitin C (*UBC*) in sacral dorsal root ganglia and spinal cord samples collected from 16 tail docked pigs (2/3rds of tail amputated) 1, 4, 8 and 16 weeks after tail injury (4 pigs/time point). Total RNA from pooled samples was measured by SYBRgreen real-time quantitative PCR. Cycle threshold values were analysed using geNorm, BestKeeper and NormFinder PCR analysis software. Average expression stability and pairwise variation values were calculated for each candidate reference gene. GeNorm analysis identified the most stable genes for normalisation of gene expression data to be *GAPDH* > *eEF-1* > *UBC* > *B2M* > *ACTB* > *SDHA* for dorsal root ganglia and *ACTB* > *SDHA* > *UBC* > *B2M* > *GAPDH* > *eEF-1* for spinal cord samples. Expression stability estimates were verified by BestKeeper and NormFinder analysis. Expression stability varied between genes within and between tissues. Validation of most stably expressed reference genes was performed by normalisation of calcitonin gene related polypeptide beta (*CALCB*). The results show similar patterns of *CALCB* expression when the best reference genes selected by all three programs were used. *GAPDH*, *eEF-1* and *UBC* are suitable reference genes for porcine dorsal root ganglia samples, whereas *ACTB*, *SDHA* and *UBC* are more appropriate for spinal cord samples.

## Highlights

• GeNorm analysis of 6 pig reference genes identified stable genes for normalisation that were verified using BestKeeper and NormFinder software
• Reference genes were variably expressed in dorsal root ganglia and spinal cord tissues
• Validation of most stably expressed reference genes was performed by normalisation of *CALCB* gene
• *GAPDH*, *eEF-1* and *UBC* genes were most stably expressed in pig dorsal root ganglia
• *ACTB* and *SDHA* were the most stably expressed pair of reference genes in pig spinal cord

## 1 Introduction

Real-time quantitative PCR (RT-qPCR) is a popular laboratory method for the measurement of gene expression in a wide variety of tissues in many different species (Bustin, 2000). In order to control and account for variations in technical processes such as differences in the quantity of starting material and sample preparation efficiency, specific “housekeeping”, reference genes, that are stably expressed in all nucleated cell types are used for normalisation (Huggett et al., 2005). Typically, the choice of normalizing genes has been based on gene studies on rodent and human tissues, however, it has become increasingly recognised that the choice of normalizing genes must be evaluated for stability in: (1) specific tissues, (2) phenotype, (3) at different stages of development and (4) effect of treatment (e.g. diseased vs. healthy tissue). Therefore, there is no single universal reference gene that is consistent in all experimental situations (Kozera and Rapacz, 2013).

A varied number of reference genes including β-actin (*ACTB*), β-2-microglobulin (*B2M*), glyceraldehyde-3-phosphate dehydrogenase (*GAPDH*), hypoxanthine phosphoribosesyl transferase 1 (*HPRT1*), peptidyl prolyl isomerase A (*PPIA*), ribosomal protein L4 (*RPL4*), succinate dehydrogenase complex subunit A (*SDHA*), TATA box binding protein (*TBP*) and tyrosine 3-monooxygenase/tryptophan 5-monooxygenase activation protein zeta polypeptide (*YWHAZ*) have previously been investigated for their expression stability in a wide range of porcine tissues including oocytes, blood, brain, muscle, stomach, intestine, skin, cartilage, bone marrow, pancreas, kidney, liver, lung, thymus, lymph nodes, spleen and heart (Kuijk et al., 2007, Nygard et al., 2007, Uddin et al., 2011, McCulloch et al., 2012), but as yet, reference gene stability has not been examined in porcine peripheral nerve dorsal root ganglia (DRG) or spinal cord (SC) tissues, nor at different ages or developmental stage.

The quantification of suitable reference genes for expression gene analysis following peripheral nerve injury after spinal nerve ligation has also been investigated in studies on rodent DRG neurons (Bangaru et al., 2012) and it is reported that reference genes exhibiting good stability included *GAPDH* and mitogen activated protein kinase 6 (*MAPK6*). Similarly, *ACTB*, *B2M*, *GAPDH* and 18S ribosomal RNA (*18S*) have also been used as suitable housekeeping genes in expression studies on wound healing in human and rodent studies (Turabelidze et al., 2010).

Tail docking (amputation of a portion of the tail) is a procedure carried out in pig production as a preventative measure against tail biting which is considered a major pig welfare issue (Nannoni et al., 2014). Tail docking, in itself, is a welfare issue causing acute inflammatory and possible chronic pain. The procedure causes complete transection of the peripheral nerves in the tail and is associated with the development of traumatic or “amputation” neuromas in the tail stump (Simonsen et al., 1991, Done et al., 2003, Herskin et al., 2015, Sandercock et al., 2016). Traumatic neuromas form at the end of the transected nerves as a consequence of non-neoplastic proliferation of neurites, Schwann and perineurial cells (Foltán et al., 2008). In humans, traumatic neuromas can be associated with increased sensitivity to touch and pain in the affected tissue or limb, caused by changes in peripheral, spinal and central neuronal function and activity (Navarro, 2009). Studying changes in neuronal gene expression is therefore essential in understanding the processes that are involved in nociception and pain processing following amputation injury (Wang et al., 2002).

In this validation study, the expression levels and stability of 6 potential reference genes were investigated using SYBR green qRT-PCR on mRNA extracted from porcine peripheral nerve DRG and SC neurons. The aim of this study was to determine the most suitable genes for normalisation of mRNA transcripts for gene expression studies on the effects of peripheral nerve injury caused by tail docking and tail biting in pigs. The genes selected for this study were chosen for their distinct cellular functions which significantly reduces the likelihood that they may be co-regulated with genes of interest associated with tissue inflammation, repair and remodelling.

## 2 Methods

All experimental procedures and animal care were approved by the Ethical Review Committees of Newcastle University and Scotland's Rural College (SRUC). The study was conducted where practicable in accordance with ARRIVE guidelines (Kilkenny et al., 2010).

### 2.1 Animals

Sixteen female pigs (*Sus scrofa domesticus*) from multiple litters (Landrace/Large White × synthetic sire line) were used from a commercial herd reared at Cockle Park Farm, Newcastle University. They were reared in loose-housing farrowing pens until weaning at 28 days then transferred to conventional weaner, grower and finisher pens until they were removed for humane killing and subsequent post-mortem tissue collection.

### 2.2 Tail docking

All the piglets used in the study were either tail docked at 3 days of age according to commercial practice using a gas-heated docking iron. Approximately two-thirds of the tail was removed from the tail docked piglets. All of the piglets were observed over the following weeks as part of routine husbandry procedures for signs of post-amputation tail infection. All docked tail stumps healed normally without complications.

### 2.3 Sedation and humane killing

Before the post mortem collection of tissues, pigs were sedated prior to humane killing. Prior to sedation, the pigs were weighed to determine the required doses for sedation and euthanasia. The pigs were sedated in a treatment pen with Stresnil (Azaperone 2 mg/kg) injected intramuscularly in the neck and monitored over a 10–15 minute period until appropriately sedated (immobile, absence of reaction to noise and tactile stimulation).

Once sedated, the ear vein was catheterized and the animal was humanely killed by injection of Euthatal (sodium pentobarbitone 150 mg/kg i.v.). Once respiratory arrest and loss of corneal reflex was confirmed, the pig was transferred a short distance (~ 5 m) to a post-mortem room. Animals were exsanguinated to confirm death before dissection. Following exsanguination the pig cadavers were plunged into a cold water bath for 5 min to arrest post-mortem metabolism. Post-mortem tissue collections were carried out 1, 4, 8 and 16 weeks after tail treatment.

### 2.4 Tissue collection

Immediately following exsanguination and cold water immersion, the pigs were quickly dissected to collect the sacro-caudal spinal cord and associated dorsal root ganglia. Once dissected out, the ensheathed spinal cord and DRG were transferred immediately into a petri dish containing ice-cold sterile RNase/DNase free phosphate buffered saline solution (Sigma-Aldrich Co. Ltd., UK) to chill and wash off any blood contamination. The spinal cord and DRG were further dissected under a stereo microscope to remove the dural membranes to obtain samples of sacral (S_1_-S_4_) spinal cord and DRG neurons for reference gene determination. All dissection equipment was carefully washed with RNaseZap (Ambion, Thermo Fisher Scientific, UK) before and after use. Immediately following final dissection all tissues were placed into sterile RNase/DNase free 2 ml cryotubes and snap frozen in liquid nitrogen. All samples were stored at − 80 °C prior to analysis.

### 2.5 Tissue RNA extractions

Sample handling, preparation and analysis were carried out following the guidelines for the Minimum Information for Publication of Quantitative Real-Time PCR Experiments (MIQE) (Bustin et al., 2009). All relevant MIQE details are either reported in the manuscript or provided by additional files.

Tissue weights were determined prior to total RNA extraction. Samples were maintained frozen on dry ice. Approximately 30 mg of spinal cord or DRG tissue was placed into 2 ml bead homogenisation tubes (Lysing matrix D - MP Biomedicals, UK) with Qiazol reagent (1 ml) to protect against RNA degradation. Tissue samples were homogenised using a FastPrep FP120 cell disrupter (Qbiogene Inc., Cedex, France) for 3 × 40 s, replacing the tubes back on ice for approximately 1 min in between homogenization bouts to prevent frictional heat build up and possible RNA degradation. The resulting homogenate was used to prepare total RNA using the RNeasy Minikit (Qiagen, UK) according to the manufacturers recommended protocol (Qiagen RNeasy Mini Kit handbook 6/2012). Preparation of RNA using this spin column purification method included a DNase digestion step to eliminate possible genomic DNA contamination.

RNA concentration and purity were determined by optical density (A_260_/A_280_ and A_260_/A_230_ ratios) using a NanoDrop 1000 spectrophotometer (Thermo Scientific, Wilmington, USA).

RNA integrity was further evaluated using a RNA Screen tape assay (Agilent 2200 TapeStation, Agilent technologies, Waldbronn, Germany). An RNA integrity number (RIN) for each sample was computed from a generated electropherogram and software. All samples exhibited intact *28S* and *18S* rRNA subunits with RIN numbers > 7 indicating minimal RNA degradation (Schroeder et al., 2006).

### 2.6 cDNA synthesis

Reverse transcriptions and qPCR reactions were performed on a Stratagene MxPro3005 qPCR system (Agilent Technologies, Waldbronn, Germany). Total RNA (1 μg) was reverse transcribed using Precision NanoScript 2 kit (PrimerDesign Ltd., Southampton, UK) according to the manufacturers protocol. Both Oligo-dT and random nonamers were used in the same reaction as an optimal priming strategy during the annealing step. RNA template (1 μl), RT primers (1 μl) and RNAse/DNase free water (8 μl) (10 μl final volume) were incubated in thin walled 0.2 ml PCR tubes (Starlab International GmbH, Hamburg, Germany) at 65 °C for 5 min, then immediately chilled on ice after the annealing process. For the extension step buffer containing: NanoScript2 4 × buffer (5 μl), dNTP mix (10 mM, 1 μl), modified Maloney murine leukaemia virus (M-MLV) enzyme (1 μl) and RNAse/DNase free water (8 μl) was added to the 10 μl of annealed sample, briefly vortexed and incubated at 42 °C for 20 min. Samples were inactivated by incubation at 75 °C for 10 min. cDNA was diluted (1:10) in RNase/DNase free-water and stored at − 20 °C until use.

### 2.7 Quantitative Real-Time RT-PCR with SYBR green

Quantitative PCR reactions were performed on a panel of 6 potential reference genes using a pig (*Sus scrofa*) geNorm™ kit with SYBRgreen detection (PrimerDesign Ltd, Southampton, UK) (Table 1). Calcitonin related polypeptide beta (*CALCB* - NM_001102473.1) primers for normalisation validation were designed using NCBI Primer-BLAST and Primer 3 from porcine gene sequences (Forward: 5′-TGCACTGGTGAAAGCCTATG-3′; bases 217–236) and (Reverse: 5′-CAGGTAGCAGTGTTGCAGGA-3′; bases 333–314) spanning one intron to detect genomic contamination and obtained from Integrated DNA Technologies (Leuven, Belgium).

**Table 1:** Candidate reference genes examined using Primerdesign 6 gene pig geNorm kit.

Gene name	Gene symbol	Accession number	Anchor nucleotide	Amplicon length (bp)	Function
Beta-actin	*ACTB*	DQ452569	189	182	Cytoskeletal protein, cell structure and integrity
Beta-2-microglobulin	*B2M*	NM_213978	78	84	Histocompatibility complex antigen
Eukaryotic elongation factor 1 gamma-like protein	*eEF1*	AF480162	163	75	Amino acylation of tRNA in ribosome (protein synthesis)
Glyceraldehyde 3-phosphate dehydrogenase	*GAPDH*	AF017079	918	188	Glycolysis, transcription and apoptosis
Succinate dehydrogenase complex subunit A	*SDHA*	DQ402993	215	100	Tricarboxylic acid cycle
Ubiquitin-C	*UBC*	M18159	122	93	Protein ubiquination (degradation, cell cycle regulation, DNA repair)

Samples (4 per post-treatment time point) were analysed in triplicate for each tissue type. The six candidate reference genes were split equally across and assayed on two 96 well plates. No RT controls were included to demonstrate there was no genomic DNA carry over. PCR amplifications of cDNA were performed in 20 μl total volume containing: resuspended primer mix (1 μl), PrimerDesign PrecisionTM 2 × qPCR Mastermix with ROX as a reference dye (10 μl), RNase/DNase free-water (4 μl), diluted cDNA (5 μl) using the following thermal cycling conditions: 50 °C for 2 min, 1 cycle at 95 °C for 10 min, followed by 40 cycles of amplification at 95 °C for 15 s and 60 °C for 1 min. The threshold cycle (C_t_) was automatically calculated by instrument software (threshold at 0.2) on the linear part of the melt curve. Raw fluorescent data (normalised reporter values, Rn values) were exported for analysis. A standard curve was obtained in a second run for each candidate reference gene using 10-fold serial dilutions of pooled cDNA (*n* = 4) to verify amplification efficiencies (Pfaffl, 2001) using the same assay conditions described above. Mean C_t_ values for each dilution were plotted against log_10_ of the cDNA input to generate efficiency plots for each candidate gene for each tissue (see Additional File 1, Additional File 2). The reaction efficiency for each gene was calculated using the equation *E* = 10^(− 1/slope)^, where *E* is the reaction efficiency and ‘slope’ is the slope of the line determined by regression analysis.

### 2.8 Analysis of reference gene expression stability

Several statistical tools are available to identify stably expressed genes (e.g. geNorm, Normfinder, Bestkeeper), although studies have shown that there is no appreciable difference between them and studies using all three tools have shown very similar outputs (McCulloch et al., 2012, Wang et al., 2012). In the current study we used the geNorm algorithm (https://www.biogazelle.com/qbaseplus) for the determination of the most stable reference genes (Vandesompele et al., 2002). Using this approach, a gene expression normalisation factor can be calculated for each sample based on the geometric averaging of multiple user-defined reference genes. The geNorm algorithm calculates the gene expression stability measure (*M*) for a reference gene as the average pairwise variation *V* for that gene with all other tested reference genes (Hellemans et al., 2007). Stepwise exclusion of the gene with the highest *M* value allows ranking of the tested gene according to their expression stability. An appropriate reference gene should have an *M* value below 0.5 or 1.0 in homogenous and heterogeneous cell/tissue sample set, respectively (Hellemans et al., 2007).

To further validate the outcomes of the GeNorm analysis the data were retrospectively re-analysed using BestKeeper (Pfaffl et al., 2004) and NormFinder (Andersen et al., 2004) software. In addition, an assessment of the effects of different gene combinations for normalisation was undertaken on a suitable gene of interest, calcitonin related polypeptide beta (*CALCB*) which codes for calcitonin gene related peptide (CGRP) a 37-amino acid neuropeptide that is a highly potent vasodilator found widely distributed in peripheral sensory neurons especially in unmyelinated C and thinly myelinated A-fibres (Russell et al., 2014).

### 2.9 Statistical analysis

The effects of time after tail treatment on candidate gene cycle threshold (C_t_) values (expression abundance) and relative fold-change expression for normalised *CALCB* C_t_ values were evaluated using a General Linear Model (GLM) ANOVA on repeated measures with sample ID as a random factor. C_t_ data are expressed as box-plots representing the 25th (Q1) and 75th (Q3) quartiles with the median line indicated within each box and whiskers showing the maximum and minimum values. All data were analysed using GenStat *for Windows* 16th Edition (VSN International Ltd., Hemel Hampstead, UK).

## 3 Results

### 3.1 PCR efficiencies

PCR amplification efficiencies of all porcine candidate reference genes for DRG and SC samples are shown in Table 2. All candidate genes exhibited efficiencies between 93 and 102% (see Additional File 1, Additional File 2 for efficiency plots) and linear regression fits (R^2^) ≥ 0.971.

### 3.2 Expression data analysis

**Fig. 1:**
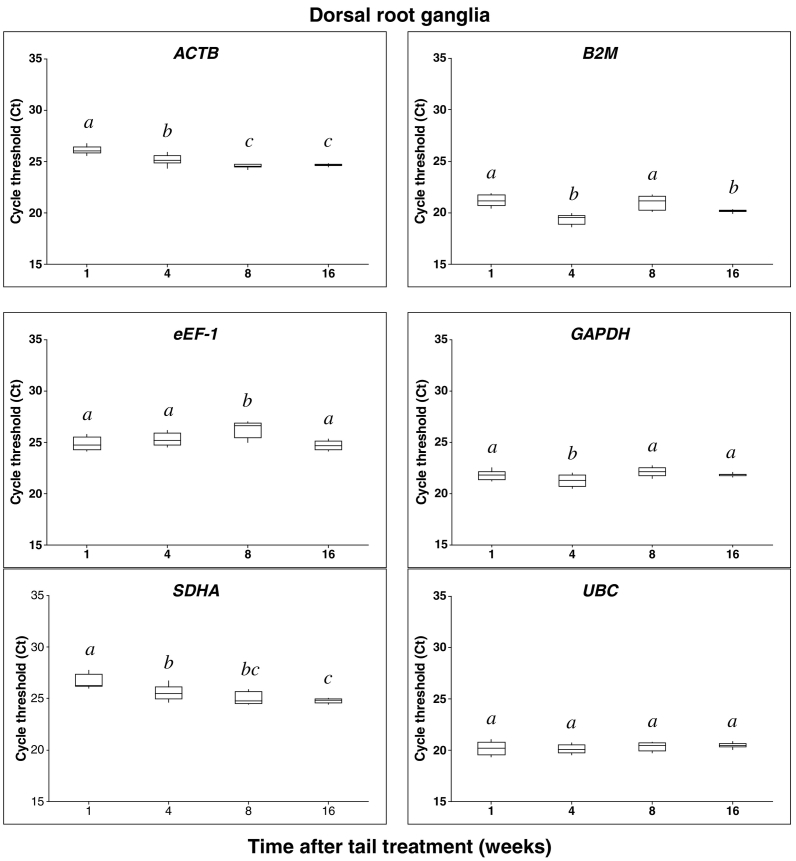
Summarized RT-qPCR cycle threshold (C_t_) values for pig sacral dorsal root ganglia (DRG) candidate reference genes. Data are expressed as box-plots representing the 25th (Q1) and 75th (Q3) quartiles with the median line indicated within each box and the whiskers showing the maximum and minimum values. RT-qPCR was run using 4 samples per time point analysed in triplicate. Groups with different superscript letters within each reference gene are significantly different (p < 0.05).

**Fig. 2:**
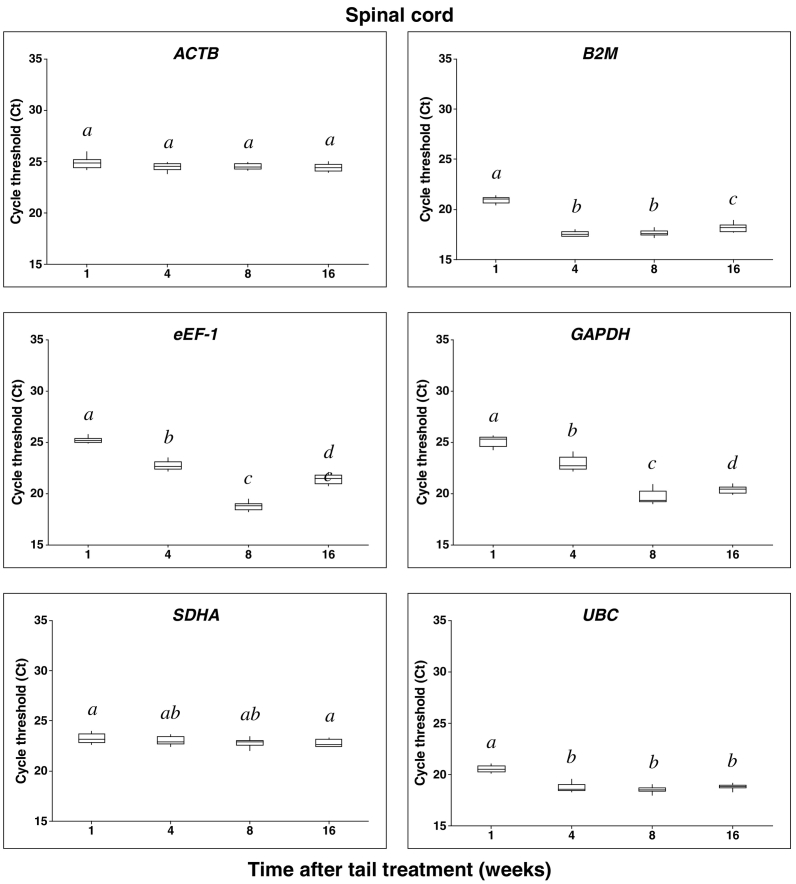
RT-qPCR cycle threshold (C_t_) values for pig sacral spinal cord candidate reference genes. Data are expressed as box-plots representing the 25th (Q1) and 75th (Q3) quartiles with the median line indicated within each box and the whiskers showing the maximum and minimum values. RT-qPCR was run using 4 samples per time point analysed in triplicate. Groups with different superscript letters within each reference gene are significantly different (p < 0.05).

PCR amplification products were obtained for all candidate reference genes at all time points after tail treatment in both DRG and SC samples, under the assay conditions described (Fig. 1, Fig. 2). The six candidate reference genes were abundantly expressed in both tissues, with median C_t_ values across all time points in DRG samples ranging from 20.29 for *UBC* as the most abundant to 25.15 for *SDHA* as the least abundantly expressed reference gene (Fig. 1). In SC samples, gene expression abundance was highest for *B2M* (17.88) and lowest for ACTB (24.53) (Fig. 2).

**Table 2:** Efficiency data for candidate normalisation genes in pooled cDNA (n = 4) obtained from pig sacral dorsal root ganglia and spinal cord samples.

	Tissue					
	Dorsal root ganglia			Spinal cord		
Gene symbol	Slope	Efficiency	*R*^2^	Slope	Efficiency	*R*^2^
*ACTB*	− 3.380	97.6	0.999	− 3.291	101.0	0.997
*B2M*	− 3.354	98.7	0.997	− 3.446	95.1	0.995
*eEF-1*	− 3.331	99.6	0.971	− 3.405	96.7	0.979
*GAPDH*	− 3.328	99.8	0.990	− 3.466	94.3	0.981
*SDHA*	− 3.495	93.3	0.996	− 3.286	101.5	0.998
*UBC*	− 3.295	101.1	0.987	− 3.345	99.4	0.995

In both neural tissue types, significant absolute C_t_ value differences were observed between each time point for nearly all reference genes except *UBC* and *ACTB* in the DRG and SC respectively (Fig. 1, Fig. 2).

### 3.3 geNorm analysis

**Fig. 3:**
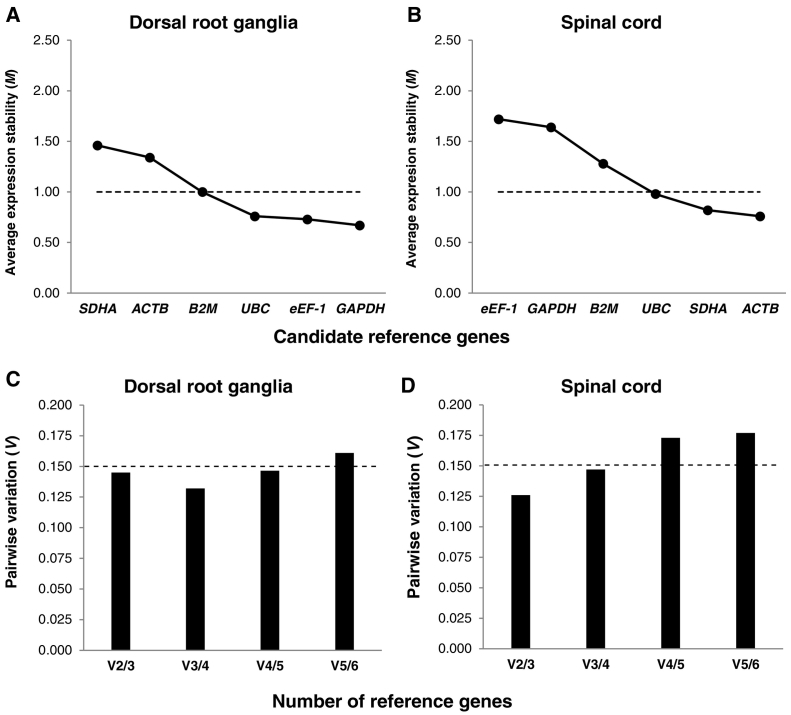
Average expression stability values (*M*) for pig sacral dorsal root ganglia (A) and spinal cord (B) samples using geNorm analysis. Candidate reference gene stability plotted from least stable (left) to most stable (right). Dashed line indicates minimum cut-off for acceptable gene stability (Hellemans et al., 2007). Pairwise variation (*V*) analysis of dorsal root ganglia (C) and spinal cord (D) candidate reference genes. *V*-score ≤ 0.15 is the recommended minimum threshold level for the optimal number of required normalization genes based on the incremental addition of genes (Vandesompele et al., 2002). RT-qPCR was run using 4 samples per time point for each tissue analysed in triplicate.

GeNorm analysis ranked the 6 candidate reference genes for each tissue type (Fig. 3A & B). For DRG samples the gene expression stability (*M*) was ranked most stable to least: *GAPDH* (0.67) > *eEF-1* (0.73) > *UBC* (0.76) > *B2M* (*1.00*) > *ACTB* (1.34) *SDHA* (1.46). For SC samples the candidate reference genes were ranked: *ACTB* (0.76) > *SDHA* (0.82) > *UBC* (0.98) > *B2M* (1.28) > *GAPDH* (1.64) > *eEF-1* (1.72).

Calculation of pairwise variation (*V*) based upon the normalisation factor (NF) values (NF_n_ and NF_n = 1_) after inclusion of the least stable reference gene indicated that the *V* value was lowest (0.132) when the 3rd most stable gene (*UBC*) was added for DRG samples (Fig. 3C), whereas the lowest *V* value (0.126) in SC samples was observed when the 2nd most stable gene (*SDHA*) was added (Fig. 3D). GeNorm pairwise variation analysis revealed that the 3 most stable reference genes identified for each tissue were below the recommended 1.5 threshold cut-off (Vandesompele et al., 2002) and would therefore be acceptable for normalisation of RT-qPCR data. On the basis of this analysis 3 genes (*GAPDH + eEF-1* *+* *UBC*) are optimal for DRG samples and 2 genes (*ACTB* *+* *SDHA*) would be optimal for spinal cord samples.

### 3.4 geNorm expression stability analysis validation

**Fig. 4:**
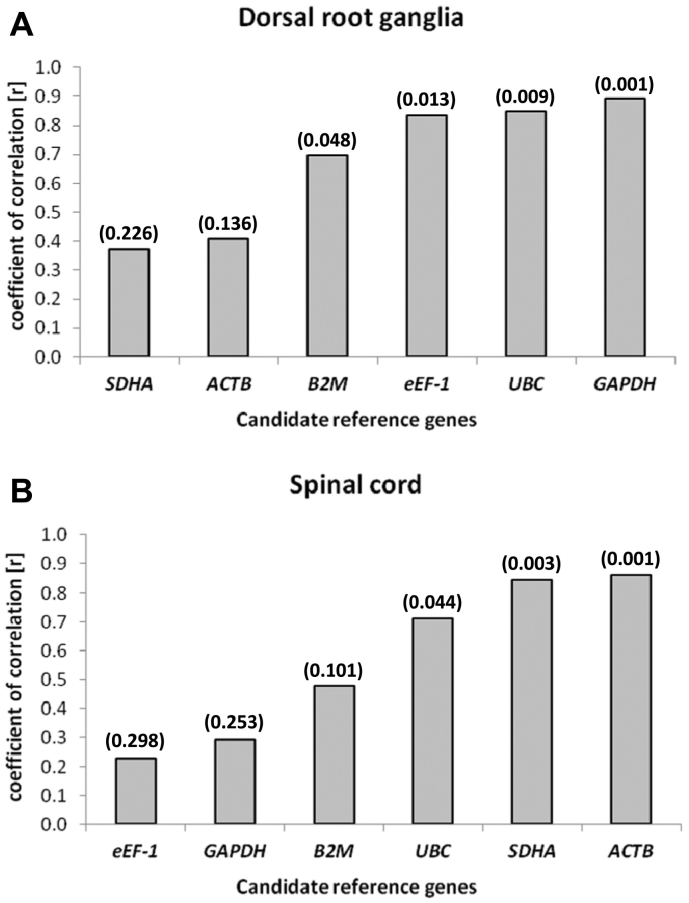
Ranked results of the candidate reference genes analysed in (A) sacral dorsal root ganglia and (B) spinal cord tissue using the BestKeeper algorithm plotted as the coefficient of correlation (r) value.  Data are ranked left to right from least to most stable expression with p value shown in parenthesis.

In order to validate estimates of stability expression by GeNorm the data were re-analysed using BestKeeper (BK) and NormFinder (NF) algorithms. The BK analysis program generates an index based on the calculation of the geometric mean of all the candidate gene C_t_ values (Pfaffl et al., 2004) and determines the stability of genes on the basis of repeated pair-wise correlation analysis one with another and with the BK index. Genes with the highest coefficient of correlation (r) are considered the most stably expressed. No specific threshold has been determined for the BK coefficients, although it is suggested that the use of multiple genes geometrically averaged is recommended to control for outliers (Pfaffl et al., 2004). Fig. 4A and B shows the stability ranking of the six candidate reference genes. *GAPDH*, *UBC*, *eEf-1* and *B2M* exhibited *r* > 0.7 for DRG neurons and *ACTB*, *SDHA* and *UBC* showed *r* > 0.7 for spinal cord. Gene ranking in both tissues by BK matched that generated by geNorm.

**Fig. 5:**
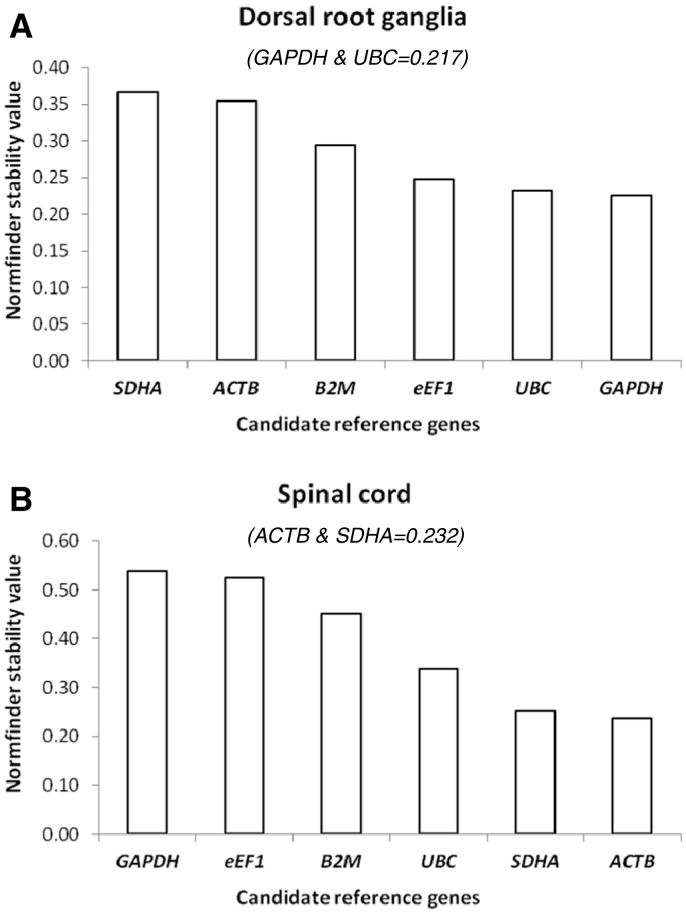
Ranked results of the candidate reference genes analysed in (A) sacral dorsal root ganglia and (B) spinal cord tissue using the NormFinder algorithm. Data are plotted as the NormFinder (NF) stability value. The best combination of two genes with their NF stability value is shown in parenthesis.

NormFinder ranks the best candidate reference genes according to their minimal combined inter and intra-group variation of expression for a normalisation factor (NF) calculation (Andersen et al., 2004). NF expression stability results are summarized in Fig. 5A (DRG) and B (SC). *GAPDH* and *UBC* were identified as the most stable genes with NF stability values of 0.226 and 0.232 respectively and provided the best combination improving the stability value of 0.217. For spinal cord *ACTB* (0.238) and *SDHA* (0.235) were identified as the most stable and the best combination with a stability value of 0.232. Candidate gene stability ranking by NF in spinal cord tissue were the same as those produced by GeNorm.

### 3.5 Reference gene validation by quantification of CALCB expression with different normalisation factors

**Fig. 6:**
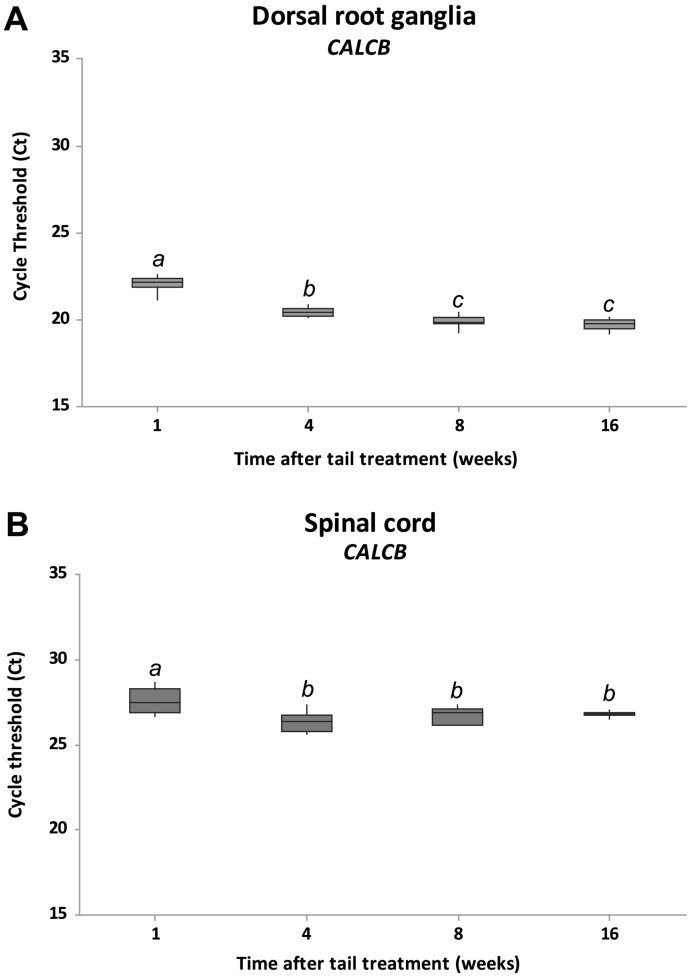
Summarized RT-qPCR cycle threshold values for *CALCB* expression in pig sacral dorsal root ganglia (A) and spinal cord neurons (B). Data are expressed as box-plots representing the 25th (Q1) and 75th (Q3) quartiles with the median line indicated within each box and the whiskers showing the maximum and minimum values RT-qPCR was run using 4 samples per time point analysed in triplicate. Groups with different superscript letters within same tissue are significantly different (p < 0.05).

Summarized C_t_ values for *CALCB* gene expression in sacral DRG and spinal cord tissues are shown in Fig. 6A and B respectively. *CALCB* gene expression was abundantly expressed in both tissues. In DRG samples there was a significant difference (*p* < 0.05) in expression dependent on age and development, although 8 and 16 weeks after treatment expression levels were not significantly different. *CALCB* C_t_ values were significantly different (*p* < 0.05) one week after tail treatment in spinal cord tissues but not after that time. In both tissues, average C_t_ values were higher (i.e. reflecting lower gene expression) in the younger pigs, suggesting age-related increases in *CALCB* transcript expression in older animals.

**Fig. 7:**
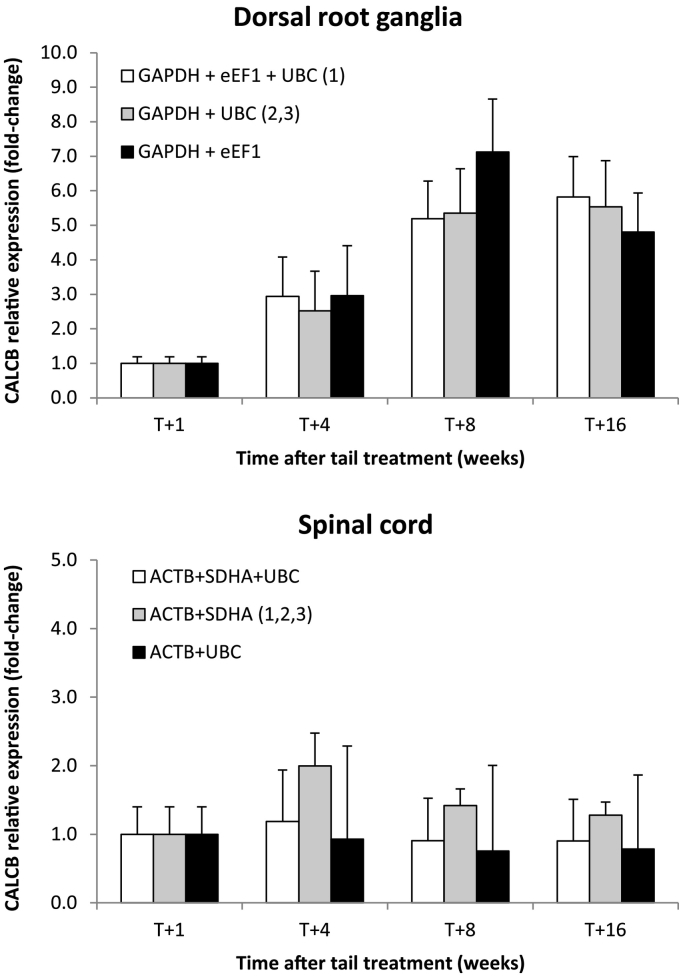
Relative quantification of *CALCB* gene in pig sacral dorsal root ganglia (A) and spinal cord (B) neurons using the calculated geometric mean of different combinations of reference genes for optimal normalisation as determined by GeNorm (1), BestKeeper (2) and NormFinder (3) software.  Data are presented as mean fold change in gene expression ± SD (n = 4 samples/time point). Fold-change expression values for T + 4, T + 8 and T + 16 weeks contrasted against T + 1 week time point.

*CALCB* C_t_ values were normalised using the geometric mean of estimated optimal combinations of reference genes as determined by GeNorm, BestKeeper and NormFinder. Fold changes in gene expression were quantified using the 2^− ΔΔCt^ method (Livak and Schmittgen, 2001). Significant fold-change increases (3–6 fold; p < 0.05) were observed in *CALCB* relative expression in sacral DRG neurons 4, 8 and 16 weeks after tail treatment compared to T + 1 week (Fig. 7A). There was however no significant difference in the quantification of *CALCB* relative expression following normalisation with the geometric mean of the different optimal combination of recommended genes determined by the three methods. In SC neurons, there was no significant change in *CALCB* relative expression over time or with different combinations of the 3 most stably expressed genes compared to the optimal combination of genes determined by the three methods (Fig. 7B).

## 4 Discussion

This is the first study to determine the stability of a number of candidate reference genes for normalisation of gene expression in porcine dorsal root ganglia and spinal cord samples. This study has identified tissue-specific groups of stable reference genes suitable for normalisation of RT qPCR data in these tissues.

As may have been anticipated, candidate reference gene expression (i.e. absolute abundance) and stability rankings varied considerably in the different neuronal tissue types. This finding is consistent with previous gene expression studies on a range of neural and non-neural porcine tissues (Nygard et al., 2007, Pierzchala et al., 2011, Timoneda et al., 2012; Uddin et al., 2011). In the present study, the most stable genes in sacral DRG samples as determined by geNorm analysis that had an acceptable *M* value < 1.00 were *GAPDH* *>* *eEF-1* *>* *UBC*, whereas in SC samples examined under the same assay conditions the most stable genes with *M* values < 1.00 were *ACTB* *>* *SDHA* *>* *UBC*. Validation of the geNorm reference gene rankings for each sample type was confirmed following re-analysis of the data using BestKeeper and NormFinder software.

The finding that *GAPDH* is the most stable transcript across all of the pig sacral DRG samples studied in our experiment is consistent with previous studies on rat lumbar (L4/L5) DRGs after spinal nerve ligation (Bangaru et al., 2012) ranking *MAPK6* and *GAPDH* by geNorm analysis as the two most stable transcripts. In contrast, in a recent spared nerve injury study on rat L4/L5 DRG, *ACTB* and *HPRT1* were the two most stable reference genes examined out of panel of seven tested that included *GAPDH* (Piller et al., 2013). Although in that study *GAPDH* was ranked lower for stability by geNorm analysis it's *M* value was well below (< 0.5) the recommended cut-off of 1.0, suggesting it was still acceptable for use in normalisation of RT-qPCR data from DRG tissue. Conversely, average *ACTB* expression stability in DRG samples in the present study was low ranked (5th out of 6).

In the present study, *ACTB*, *SDHA* and *UBC* were ranked highest by geNorm analysis as the three most stable genes for pig spinal cord. *ACTB* and *SDHA* were also ranked the most stably expressed and optimal combination by NormFinder and had the highest coefficient of correlation (*r*) values determined by BestKeeper. As this is the first study (as far as we are aware) to validate reference gene expression in porcine spinal tissue it is not yet possible to directly compare these findings with previous porcine studies on the same tissue, although *ACTB*, *RPL4*, *TBP* and *HPRT1* have previously been reported as good reference genes across a range of tissues, including neural tissues, such as cerebral cortex and hippocampus (Nygard et al., 2007). The results of the present study agree, in part, with findings from a recent study on rat lumbar spinal cord dorsal horn (Piller et al., 2013) where the three most stable reference genes by determined by geNorm analysis and stepwise elimination were *ACTB* and ribosomal proteins L29 and L13a (*RPL29*, *RPL13a*). The ribosomal genes were not available as part of the PrimerDesign porcine geNorm reference gene panel, but may be investigated in future studies as possible reference genes in porcine spinal tissues. The findings of the present study serve to emphasise that there is considerable variation in reference gene expression in different tissue types within species and that it is essential for accurate quantification of RT-qPCR data that a priori validation studies are carried out in order to determine the most suitable reference genes for a given tissue. It has been recommend by Timoneda et al. (2012) based on an extensive study on reference microRNAs (miRNA) in wide range of porcine tissues that at least five reference miRNAs should be used in studies of multiple tissues, and three in tissue-specific studies.

In the present study, marked differences in absolute transcript abundance were observed for time after tail treatment for all candidate reference genes in both neuronal tissue types, except for *UBC* in the DRG samples. As these samples were derived from sacral and not caudal tissues it is unlikely that these differences are attributable to tail treatment, although this cannot be entirely discounted. The observed differences are more likely to be due to developmental or age-related changes in reference gene expression (Touchberry et al., 2006). The time points in the current study correspond to different stages of development in the pigs namely early post natal (+ 1 week), late post natal (+ 4 weeks), pre-pubertal (+ 8 weeks) and peri-pubertal/adult (+ 16 weeks). Typically, in both neuronal tissues, absolute transcript expression tended to be lowest in samples from the earliest time point and as such may reflect possible developmental differences in gene expression. While it is not possible presently to directly compare the findings of the current study with similar studies on pig neural tissues, previous research by Uddin et al. (2011) on reference genes measured in a wide variety of different tissues obtained from neonatal, juvenile and adult pigs revealed that collectively *RPL4*, *PPIA* and *YWHAZ* were the most stable genes across all ages as determined by geNorm, NormFinder and BestKeeper analysing methods. Although no neural tissues were investigated in the aforementioned study, their analyses showed that *GAPDH* was the least stably expressed gene across the different pig ages and tissues, which is consistent to some extent with the geNorm analysis of the SC samples in which *GAPDH* was ranked 5 or 6 out of 6 for relative expression stability in the present study by the three different assessment methods.

It is recognised that reference gene expression may vary between normal healthy and diseased, infected, injured or malignant tissues (Kozera and Rapacz, 2013). The tissues obtained in the current study were obtained from pigs that had undergone tail docking during the early neonatal period. At the time of tissue collection, both caudal and sacral tissues were obtained, although subsequent reference gene RT-qPCR analysis was only performed on RNA isolates from the sacral tissues due to the limited amount of RNA available after processing the caudal tissues. Therefore it is not possible on the basis of these data to confirm or otherwise a priori if tail injury by tail docking alters expression patterns in the 6 candidate reference genes examined in this study in the caudal tissues. Studies on rodent models of cutaneous wound healing and tissue regeneration (Turabelidze et al., 2010) have reported contrasting reference gene expression stability (geNorm) in wounded (full-thickness dermal punch biopsy) and unwounded tissues. In normal skin *UBC*, *SDHA* and Cytochrome c-1 (*CYC1*) were stably expressed over time, however in healing tissue expression profiles varied in the first 24 h of injury with *TBP*, *B2M* and *RPLP2* being the most stable and *TBP*, *B2M* and *GAPDH* five days after injury. Recent studies on rat models of neuropathic and inflammatory pain have separately reported that the most stably expressed genes reported in the respective studies in brain, spinal and dorsal root ganglia tissues did not appear to exhibit any treatment induced time-dependent regulation in reference gene expression (Bangaru et al., 2012, Piller et al., 2013, Walder et al., 2014).

Since there is no universal accepted method for the determination of reference gene expression stability in the first instance we opted to evaluate six well known candidate genes using a pre-existing gene panel provided by Primerdesign with its associated geNorm software for gene stability analysis (Vandesompele et al., 2002). In order to validate the geNorm findings we retrospectively re-analysed the gene expression data using two different computer programs BestKeeper (Pfaffl et al., 2004) and NormFinder (Andersen et al., 2004).

Rankings of candidate reference gene expression stability were in concordance across the three methods for the genes used in this study. For DRG neurons, *GAPDH*, *eEF-1* and *UBC* were always ranked the most stable across methods and *ACTB*, *SDHA* and *UBC* the same for SC samples. These results suggest that there was good agreement on the determination of the most stable reference genes provided by the three different approaches in this study; however it is recommended that the evaluation of reference genes should always be cross-validated across more than one assessment method for every set of samples under different experimental conditions before use.

In order to examine the effects of different combinations of reference genes for normalisation of a specific gene of interest, the RT-qPCR expression profile of *CALCB* was determined in both sample groups. Quantification of expression was performed by a relative approach using the 2^(− ΔΔCt)^ method (Livak and Schmittgen, 2001) with normalisation against the geometric mean of the most stable gene combinations. *CALCB* transcript was highly expressed in both sample groups, but more so in DRG than in SC neurons (group average C_t_ 22 vs. 27 respectively) and its abundance marginally increased in both tissue groups with age. It is unlikely that this increase is caused by tail treatment as the only the caudal DRG neurons and associated dorsal SC neurons are affected by tail amputation, although it is acknowledged that CGRP is significantly upregulated in response to nerve damage, such as peripheral axotomy and that the synthesis of this peptide is enhanced in tissues that are undergoing and inflammatory response (Donnerer and Stein, 1992).

Investigation of different combinations of reference genes revealed no difference in *CALCB* relative expression between the three different assessment methods in either of the tissue groups. While this suggests there was good agreement in the determination of optimal genes for normalisation in this sample set, it is recognised that where gene expression levels vary considerably or are very low or high compared to reference gene expression that this may affect the normalisation results and again demonstrates the need to fully evaluate the stability of reference genes across multiple assessment platforms before use on sample sets.

## 5 Conclusions

Using three different methods of analysis (geNorm, BsetKeeper and NormFinder) we have validated a set of six candidate reference genes for gene normalisation using RT-qPCR in porcine dorsal root ganglia and spinal cord tissues. Gene expression stability values differed in the two tissues but within tissues were in concordance across the three methods.

Optimal gene combinations for normalisation were similar across the three methods and validated against *CALCB* expression in both tissues. Based on the findings of this preliminary study it is suggested that the three most suitable reference genes for normalisation calculations are *GAPDH*, *eEF-1* and *UBC* for dorsal root ganglia and *ACTB*, *SDHA* and *UBC* for spinal cord tissues. These results constitute the starting point for selecting reference genes in future mechanistic pain studies on tail docking and tail biting in pigs.

The following are the supplementary data related to this article.

Additional File 1 Mean cycle threshold (Ct) values for each dilution plotted against log_10_ of the cDNA input for each candidate gene. Additional File 1

Additional File 2 Mean cycle threshold (Ct) values for each dilution plotted against log_10_ of the cDNA input for each candidate gene. Additional File 2

## Conflicts of interest

None.

## Author's contributions

DS and SE conceived the study design. DS and PDG performed the on-farm experimental procedures and post mortem tissue collection. JC prepared RNA and cDNA from the post mortem samples and conducted the RT-qPCR and geNorm analysis and collated and summarized the data. DS carried out data analysis, interpretation of the data and drafted the manuscript. SE provided critical revision of the manuscript. All authors have read and approved the final manuscript.
