# Supplementary material for: Determination of stable reference genes for RT-qPCR expression data in mechanistic pain studies on pig dorsal root ganglia and spinal cord
Source: Res Vet Sci. 2017 Oct;114:493–501. doi: 10.1016/j.rvsc.2017.09.025 (PMC5667896; doi:10.1016/j.rvsc.2017.09.025)
Supplement: Additional File 1 — Mean cycle threshold (Ct) values for each dilution plotted against log10 of the cDNA input for each candidate gene. [file mmc1.docx]

**RT-qPCR Efficiency Plots**

**Dorsal root ganglia**

| 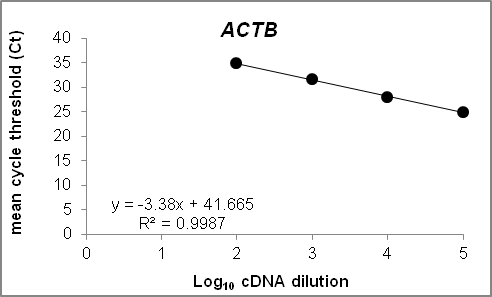 | 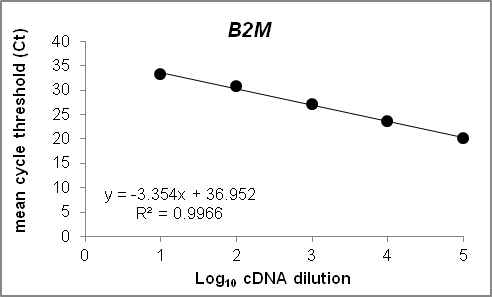 |
| --- | --- |
| 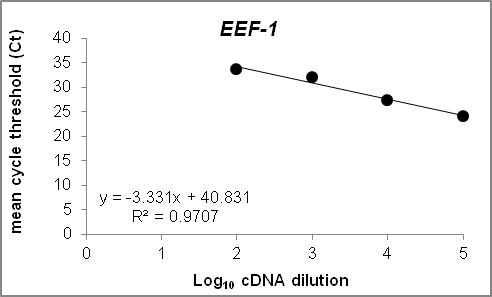 | 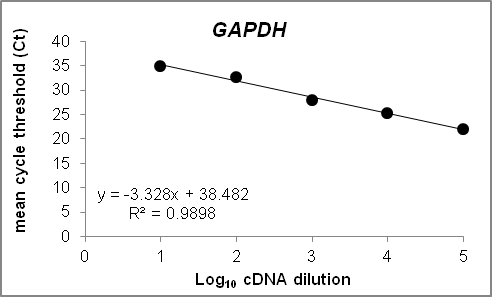 |
| 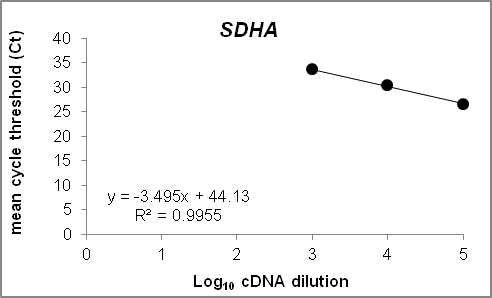 | 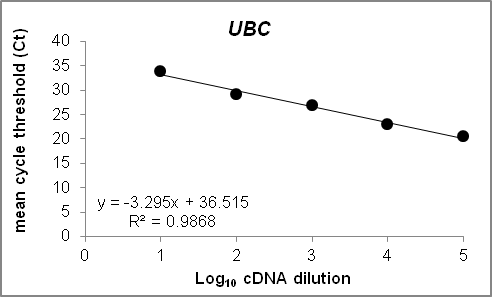 |

**Additional File 1**. Mean cycle threshold (Ct) values for each dilution plotted against log_10_ of the cDNA input for each candidate gene.
